# Fabrication of PbO_2_/PVDF/CC Composite and Employment for the Removal of Methyl Orange

**DOI:** 10.3390/polym15061462

**Published:** 2023-03-15

**Authors:** Laizhou Song, Cuicui Liu, Lifen Liang, Yalong Ma, Xiuli Wang, Jizhong Ma, Zeya Li, Shuqin Yang

**Affiliations:** 1 Hebei Key Laboratory of Heavy Metal Deep-Remediation in Water and Resource Reuse, School of Environmental and Chemical Engineering, Yanshan University, Qinhuangdao 066004, China; 2 Department of Environmental Engineering, Hebei University of Environmental Engineering, Qinhuangdao 066102, China

**Keywords:** dyeing wastewater, in situ electrochemical oxidation, lead dioxide/polyvinylidene fluoride/carbon cloth composite, methyl orange, ammonium

## Abstract

The in situ electrochemical oxidation process has received considerable attention for the removal of dye molecules and ammonium from textile dyeing and finishing wastewater. Nevertheless, the cost and durability of the catalytic anode have seriously limited industrial applications of this technique. In this work, the lab-based waste polyvinylidene fluoride membrane was employed to fabricate a novel lead dioxide/polyvinylidene fluoride/carbon cloth composite (PbO_2_/PVDF/CC) via integrated surface coating and electrodeposition processes. The influences of operating parameters (pH, Cl^−^ concentration, current density, and initial concentration of pollutant) on the oxidation efficiency of PbO_2_/PVDF/CC were evaluated. Under optimal conditions, this composite achieves a 100% decolorization of methyl orange (MO), 99.48% removal of ammonium, and 94.46% conversion for ammonium-based nitrogen to N_2_, as well as an 82.55% removal of chemical oxygen demand (COD). At the coexistent condition of ammonium and MO, MO decolorization, ammonium, and COD removals still remain around 100%, 99.43%, and 77.33%, respectively. It can be assigned to the synergistic oxidation effect of hydroxyl radical and chloride species for MO and the chlorine oxidation action for ammonium. Based on the determination of various intermediates, MO is finally mineralized to CO_2_ and H_2_O, and ammonium is mainly converted to N_2_. The PbO_2_/PVDF/CC composite exhibits excellent stability and safety.

## 1. Introduction

Textile dyeing and finishing wastewater is considered to be one of the major pollution sources to received water bodies, in terms of the tremendous discharge of dye pollutants [1]. Among the dyes, annually, the azo dye has been extensively employed and accounts for approximately 70% of the dye products [2]. As for azo-dye-including wastewater, it exhibits explicit characteristics of significant stability, intensive chroma, high biotoxicity, and refractory biodegradation due to the presence of azo functional groups (-N=N-) and aromatic rings, ultimately triggering aesthetic problems and negative effects on aquatic creatures [3]. Furthermore, reagents of urea and ammonium are required in the partial textile process, which are the main reasons for the existence of a high concentration of ammonium nitrogen (NH_4_^+^-N) in effluent, which causes eutrophication pollution and exerts serious toxicity to human beings [4,5]. For the emission requirement of dye wastewater to newly built enterprises, the Chinese Wastewater Pollutants Discharge Standard for Textile Printing Industry (GB Synthesis 87-2012) regulates the threshold values of major pollutant indexes for direct emission as follows: chroma ≤ 50, chemical oxygen demand (COD) ≤ 80 mg L^−1^, NH_4_^+^-N ≤ 10 mg L^−1^, total nitrogen (TN) ≤ 15 mg L^−1^, and pH = 6–9 [6]. Undoubtedly, the threshold limits mentioned above are a tremendous environmental load for receiving surface water, with respect to the minimum requirements (COD ≤ 40 mg L^−1^, NH_4_^+^-N and TN ≤ 2 mg L^−1^, and zero for chroma), according to the Chinese Environmental quality standards for surface water (GB 3838-2002) [7]. Thus, it is imperative to treat the produced dye wastewater before the drainage to surface water; particularly, serious attention should be given to COD, chroma, NH_4_^+^-N, and TN.

Up until now, various chemical, biological, and physicochemical techniques have been employed to treat wastewater containing pollutants of dyes and NH_4_^+^-N, but their applications are limited as being applied individually [8,9,10,11]. For instance, the physicochemical techniques such as adsorption-precipitation and chemical/photodegradation may generate secondary pollution or require post-treatment [12], and the biological processes are not efficient at degradation owing to the biodegradability recalcitrant of the dye molecules [13]. By contrast, the advanced oxidation systems (homogeneous and heterogeneous photocatalytic degradation, Fenton oxidation, persulfate oxidation, ozone oxidation, electrochemical oxidation, and combined setups of them) are competent for decoloring and mineralizing dyes via the oxidation of active hydroxyl radicals (•OH) or other free radicals (such as SO_4_^•−^ and O_2_^•−^) [14], but the response for the removal of ammonium is undesirable. Superior to free radical species, the active chlorine is competent for the removal of ammonium [15]; of course, the structures of dye molecules can be destroyed by active chlorine [16]. However, for the generally active chlorine-based oxidation systems (liquid chlorine, sodium hypochlorite (NaClO)), the transportation and storage will lead to serious environmental risks [17,18]. For the chemical oxidation techniques, to eradicate the potential environmental problems linked to transportation and storage, an alternative strategy (i.e., in situ electrochemical generations of oxidants) will be a commendable choice. In the electrochemical oxidation (EO) process, active radical species (•OH, SO_4_^•−^, and O_2_^•−^) are produced on the anode surface; the chloride ion (Cl^–^) is electrochemically oxidized to the chlorine molecule (Cl_2_), and then part of Cl_2_ consequently dissolves in solution to produce other active chlorine species (hypochlorous acid (HClO) in neutral and acidic solutions and hypochlorite ions (ClO^–^) in alkaline solution) [19,20,21,22]. The aforesaid active species have been validated to remove the dye-type and other organic pollutants from the solution [23,24,25,26]. Unlike •OH, SO_4_^•−^, and O_2_^•−^ species, the species of active chlorine (Cl_2_, HClO, and ClO^–^) can efficiently remove the ammonium besides the organic pollutants [15,27,28,29,30,31].

A variety of electrodes have been successfully applied to remove dye and ammonium contaminants via the electrochemical oxidation processes. For instance, methyl orange can be effectively removed by the Ti_4_O_7_ anode oxidation [28], with COD removal up to 91.7% after electrolysis for 5 h. Kim et al. [29] compared the electrolysis efficiencies of ammonium by IrO_2_, RuO_2_, and Pt electrodes, and there are no essential differences among them for the removal of ammonium, which attests that the addition of chloride ions is favorable to ammonium removal. The oxidation of active chlorine generated by the Ti/IrO_2_-RuO_2_ electrode on the removal of ammonium in the presence of Cl^–^ has also been illustrated by Zhang et al. [30], and the removal of NH_4_^+^-N at 50 mg L^−1^ can reach 95% with the main end-product of N_2_. Furthermore, Mandala et al. [31] obtained a similar result for the removal of ammonium using a relatively low-cost G/PbO_2_ electrode. However, the above electrochemical oxidation reports mainly aimed at the removal of a single dye molecule or NH_4_^+^ from the solution. With respect to the coexistence of dye molecules and ammonium in dyeing wastewater, in situ electrochemical generations of free radicals and active chlorines should be needed. To the best of our knowledge so far, sufficient efforts focusing on this issue are unavailable. BDD, as a typical inactive electrode, applied generally for the electrochemical oxidation process in contrast to active anodes of transition metal oxides (TMOs), generates higher hydroxyl radicals, and exhibits a higher ammonium removal efficiency in the absence of chlorine. However, in the case of nonexistent Cl^−^, the PbO_2_ electrode is more advantageous due to the higher electrogeneration of active chlorine.

Compared with other functional anodes (such as Ti/IrO_2_-RuO_2_), the PbO_2_ electrode has attracted tremendous attention due to its high conductivity, low cost, and high hydroxyl radicals (•OH) generation capability [32]. In terms of the engineering perspective, besides the dissolution of lead ions, the corrosion destruction of the PbO_2_ electrode caused by active chlorine cannot be ignored. To facilitate the practical application of the PbO_2_ electrode, the polymer of polyvinylidene fluoride (PVDF) was reported [33] to incorporate the framework PbO_2_ of microparticles by means of an anodic electrodeposition process; however, the incorporation of PVDF reduced the electrical conductivity and active sites of the fabricated PbO_2_ anode.

In this work, we aim to tune the incorporation strategy of PbO_2_ microparticles into the PVDF framework to guarantee the concerns of thimbleful dissolution for lead, excellent durability, and in situ, efficient electrochemical generation of free radicals and active chlorine. Thus, a PbO_2_/PVDF/CC composite anode was fabricated by means of facile surface coating and galvanostatic electrodeposition processes. The morphology, crystal phase, and chemical composition of the composite were characterized by X-ray diffractometry (XRD), field emission scanning electron microscopy (SEM), and X-ray photoelectron spectroscopy (XPS). The influences of key parameters (pH value, initial Cl^−^ concentration, current density, and initial concentration of dye) on the electrochemical oxidations of ammonium and methyl orange via hydroxyl radicals and active chlorine were systematically studied. The reusability and lead dissolution of the fabricated composite were further assessed. Finally, the mechanism of decolorization and mineralization of methyl orange (MO) dyes and the chlorine oxidation process of ammonium were elaborated.

## 2. Experimental Sections

### 2.1. Materials

Detailed information of employed reagents is presented in the Supplementary Material, and they were used as received.

### 2.2. Fabrication of PbO_2_/PVDF/CC Composite

#### 2.2.1. Preparation of PVDF/CC Substrate

The CC sample (2.0 cm × 2.0 cm) needed to remove greasy dirties or impurities on the surface prior to use, and the pretreatment process is reported in the Supplementary Material. The employed polyvinylidene fluoride (PVDF) polymer was the lab-based waste PVDF membrane; before usage, it was washed using ethanol and deionized water and then dried in a drying oven at 60 °C overnight. The fabrication process of the PVDF/CC sample is described as follows. First, powders of CaCO_3_, acetylene black, and PVDF with a ratio of 1:1:0.8 (*w*/*w*) were vigorously blended; NMP was employed as the solvent with a dosage of 0.3 mL/0.028 g mixture; the mixture was fully ground in a mortar to form a homogeneous slurry. After that, the obtained slurry was coated on the surface of the pretreated CC, and the coated CC sample was desiccated at 50 °C for 10 h. The obtained sample thoroughly leached the incorporated CaCO_3_ powders with 4 mol L^−1^ of hydrochloric acid solution and then washed with deionized water and ethanol for several cycles. Eventually, the PVDF/CC sample was dried overnight in an oven at 60 °C. For comparison, the sample without the addition of CaCO_3_ particles (PVDF/CC-1) was also prepared under the same conditions.

#### 2.2.2. Preparation of PbO_2_/PVDF/CC Composite

**Scheme 1:**
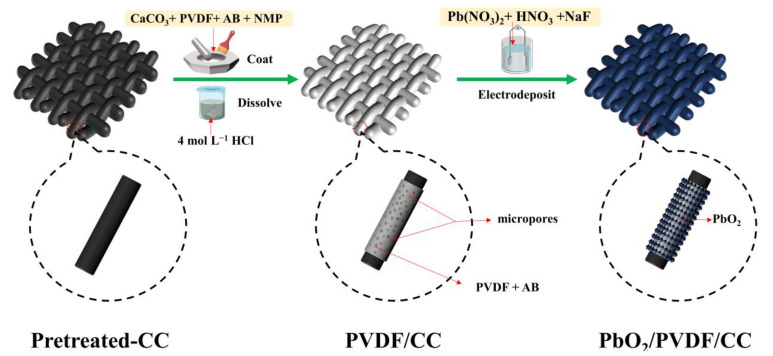
Schematic diagram for the fabrication of PbO_2_/PVDF/CC composite. Notes: AB: acetylene black, NMP: N-methyl-2-pyrrolidinone, CC: carbon cloth, PVDF: polyvinylidene fluoride.

For the fabrication of the PbO_2_/PVDF/CC composite, the PbO_2_ active layer was deposited on the PVDF/CC substrate surface by an anodic electrodeposition process, where the plating solution consisted of 0.5 mol L^−1^ of Pb(NO_3_)_2_, 0.1 mol L^−1^ of HNO_3_, and 0.5 g L^−1^ of NaF. The PVDF/CC substrate served as the anode while the Ti plate substrate acted as the cathode, and their immersed areas and the distance between them were set as 1 × 2 cm^2^ and 1.5 cm, respectively; the volume of the anolyte was 50 mL. Subsequently, the electrodepositing experiment was performed for 15 min, using a ZF-9 Potentiostat (Zhengfang Electronics Co., Ltd., Shanghai, China) to provide the current density of 40 mA cm^−2^. During the electrodeposition process, the solution was magnetically stirred at a rate of 100 rpm. After that, the sample was rinsed with deionized water before being dried in a vacuum drying oven. As a result, the surface of the PVDF/CC sample was covered by a thin film of navy blue β-PbO_2_ with an average deposition amount of 62 mg cm^−2^. Four other PbO_2_/PVDF/CC composites were also prepared by the use of a similar procedure mentioned above by adjusting the deposition time (5, 10, 20, and 25 min). The preparation procedure of the PbO_2_/PVDF/CC composite is illustrated by Scheme 1. For the purpose of comparison, the PbO_2_/CC sample was also prepared using a similar process to that of PbO_2_/PVDF/CC with CC in place of the PVDF/CC substrate.

### 2.3. Characterization

#### 2.3.1. Conventional Characterization

The morphologies of PbO_2_/CC, PVDF/CC, and PbO_2_/PVDF/CC composites were monitored by a scanning electron microscope (FE-SEM, SUPRA 55, Zeiss, Jena, Germany) with an accelerating voltage of 20 kV, and an energy-dispersive X-ray analyzer (EDX) was coupled into this microscope to determine the chemical elements of the tested samples. The crystal structures and phase constitutions of fabricated samples were analyzed using an X-ray diffraction meter (XRD, Smart Lab, Tokyo, Japan) at a scan rate of 5° min^−1^ with Cu Kα radiation (*λ* = 1.54178 Å), with the scanning angle (2*θ*) ranging from 10° to 90°. The surface chemical components and element valence states of the obtained samples were evaluated by an X-ray photoelectron spectroscope (XPS, Escalab 250, Thermo Fisher Scientific, Waltham, MA, USA) with Mg Kα as an excitation source. The C 1s peak (284.8 eV) was used to calibrate the binding energies.

#### 2.3.2. Electrochemical Characterizations

The electrochemical measurements were performed on an electrochemical workstation (CHI 650C, Chenhua Co., Ltd., Shanghai, China) in a three-electrode cell system with the as-synthesized sample as the working electrode, and the platinum sheet and Ag/AgCl (saturated KCl solution) electrode as the counter electrode and the reference electrode, respectively. The linear sweep voltammetry (LSV) for the electrochemical chlorination reaction (CER) over the prepared electrodes was measured in 5.0 mol L^−1^ of NaCl solution at the scan rate of 0.002 V s^−1^ with the voltage ranging from 0 to 1.6 V (vs. Ag/AgCl). The calculation of the double-layer capacitance (*C*_dl_) values and the electrochemical active surface areas (ECSA) was detailed in the Supporting Information. The oxygen evolution reaction (OER) polarization experiments were performed in 0.5 mol L^−1^ of H_2_SO_4_ solution at the scan rate of 0.01 V s^−1^, and the voltage window was 0–2.5 V (vs. Ag/AgCl).

### 2.4. Electrochemical Oxidation Process

The electrochemical oxidation processes of ammonium and MO were conducted in an undivided electrolytic cell with a 200 mL solution containing NH_4_^+^ or MO as the target pollutant, and 0.05 mol L^−1^ of Na_2_SO_4_ as the supporting electrolyte was used to retain the conductivity. The as-synthesized electrode and the graphite plate with the same effective area of 2 cm^2^ were employed as the anode and cathode, respectively, and the distance between them was 1.5 cm. Initial pH values of the electrolyte solutions were adjusted to the target data using 0.5 mol L^−1^ NaOH or 0.5 mol L^−1^ H_2_SO_4_ solutions. The electrochemical treatment procedures were performed under galvanostatic conditions with the ZF-9 potentiostat to provide constant anode current. Considering that NH_4_^+^ and MO influence each other and the removal issue of NH_4_^+^ by active chlorine, we selected NH_4_^+^ as the main target pollutant to investigate the efficiency of the chlorine oxidation process. Batch experiments were carried out to ascertain the optimal operation parameters on the oxidation efficiency of chlorine (pH, current density, initial concentration of contaminants, and initial Cl^–^ concentration). Each electrochemical test was conducted in duplicate for the single MO oxidation system and the mixture oxidation system of NH_4_^+^ and MO. During the experiments, some solutions were taken out at scheduled time intervals to measure existing concentrations of NH_4_^+^-N (Nessler’s reagent spectrophotometry, HJ 535-2009), NO_3_^−^-N (UV spectrophotometry, HJ/T 346-2007), NO_2_^−^-N (N-(1-naphthol) ethylenediamine spectrophotometry, GB 7493-87), free chlorine, NH_2_Cl, NHCl_2_, NCl_3_, and MO via a UV-vis spectrophotometer (UV-240, Shimadzu, Kyoto, Japan) according to N,N-diethyl-1,4-phenylenediamine spectrophotometry (HJ 586-2010). The pH value was measured by a pH meter (PHS-2F, Yidiankexue, Co., Ltd., Shanghai, China). In addition, the mineralization of MO was monitored from the abatement of the TOC measured on a total organic carbon analyzer (TOC-V CHP, Shimadzu, Japan). The chemical oxygen demand (COD) was estimated by the Chinese standard dichromate method (GB11914-1989). Meanwhile, the masking experiment of free radicals was conducted under optimal reaction conditions as mentioned in the PbO_2_/PVDF/CC system, where TBA was used as a hydroxyl radical scavenger to analyze the residual •OH. The effect of different concentrations of TBA on the degradation rate of MO was studied to identify the type and role of active species in the system. In addition, high-performance liquid chromatography coupled with a mass spectrometer (HPLC-MS Agilent 6460 Triple Quadrupole MS, Santa Clara, CA, USA) was used for analyzing the degradation byproducts of MO. An atomic absorption spectrophotometer (WFX-120, Ruili, Co., Ltd., Beijing, China) was used for the analysis of lead concentration, thereby evaluating the stability and environmental security of the fabricated PbO_2_/PVDF/CC composite. The calculations of removal efficiencies for NH_4_^+^, decolorization, TOC, COD, and TN are shown in the Supporting Information.

## 3. Results and Discussion

### 3.1. Characterizations of PbO_2_/PVDF/CC Composite

**Figure 1:**
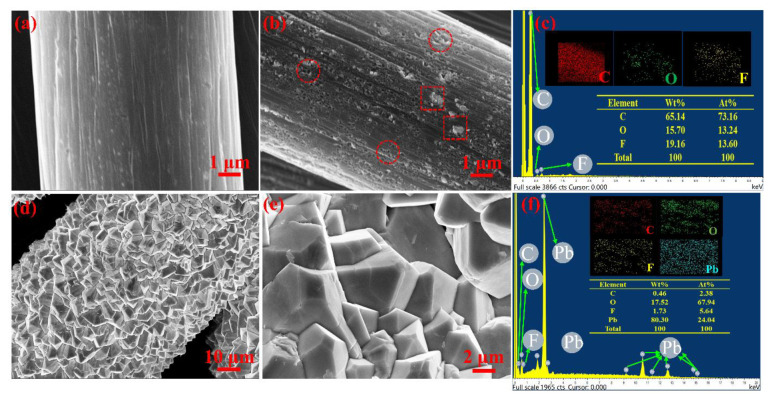
SEM and EDX images: (**a**) CC; (**b**) PVDF/CC; (**c**) EDX spectrum of PVDF/CC; (**d**,**e**) PbO_2_/PVDF/CC at different magnifications; (**f**) EDX spectrum of the PbO_2_/PVDF/CC electrode.

The morphologies of CC, PVDF/CC, and PbO_2_/PVDF/CC composites are observed via SEM (Figure 1). The surface of the pretreated CC is smooth and without obvious impurities (Figure 1a). Compared to CC, the PVDF/CC sample exhibits a different morphology, as displayed by Figure 1b; a layer of PVDF polymer is anchored on the surface of CC, in line with the results of the EDX test (Figure 1c). It is exciting that the PVDF particles (tabbed by red squares) and lots of concave pores (tabbed by red circles) are discovered on the surface of CC. The presence of PVDF particles derived from the dissolution of CaCO_3_ will be helpful to increase the surface roughness of the substrate and can then be beneficial to the subsequent deposition of the PbO_2_ active layer. In addition, no Ca element can be detected (Figure 1c), confirming that the CaCO_3_ powder is completely dissolved by hydrochloric acid solution. The SEM image of PVDF/CC-1 is also monitored (Figure S1a), and it can be clearly seen that the carbon fibers tightly link to each other and the PVDF film magnificently covers the surfaces of CC, which is unfavorable to the subsequent deposition of the PbO_2_ active layer.

After anodic deposition, the PbO_2_ microparticles (Figure 1d) are tightly bounded to the surface of the PVDF/CC sample, and the formation of a flake-like thin PbO_2_ layer is readily identified. It can be observed that the deposited nanoparticles are pyramid-shaped (clearly shown in Figure 1e), and the results of the corresponding EDX (Figure 1f) further reveal that the anchored layer is mainly composed of Pb and O elements; a relatively small amount of F element is also detected due to the residual NaF. The presence of the PVDF layer could make the crystalline grain of PbO_2_ compact, thus contributing to improving the durability of the fabricated composite. The SEM image of the PbO_2_/CC electrode used for comparison is displayed in Figure S1b, and those of PbO_2_/PVDF/CC samples with different electrodeposition times (5, 10, 20, and 25 min) are displayed in Figure S2a–d. It can be observed that the mean microcrystal sizes of PbO_2_ are 2.30 and 2.65 μm at electrodeposition times of 5 and 10 min, smaller than those at 20 and 25 min (5.02 and 6.71 μm), respectively. The shorter electrodeposition time is undesirable to form pyramid-shape PbO_2_ microparticles, but the prolongation of electrodeposition time leads to the appearances of large grains, nodulation, and an unsmooth surface, and the mean microcrystal size of PbO_2_ at 15 min is 4.75 μm; thus, 15 min serves as the optimal electrodeposition time.

**Figure 2:**
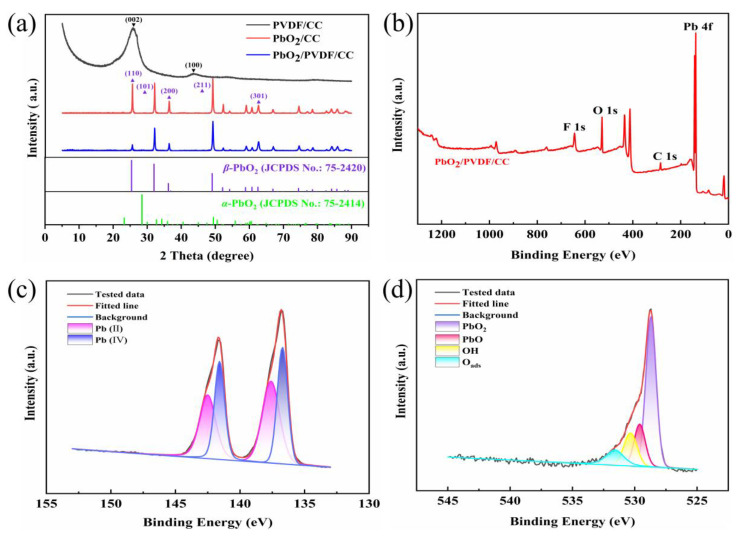
XRD and XPS spectrum: (**a**) XRD patterns of PVDF/CC substrate and PbO_2_/PVDF/CC composite; (**b**) XPS fully scanned spectra of PVDF/CC and PbO_2_/PVDF/CC; (**c**) Pb 4f XPS spectrum of PbO_2_/PVDF/CC composite; (**d**) O 1s core level spectrum for PbO_2_/PVDF/CC electrode.

The XRD patterns of PVDF/CC, PbO_2_/CC, and PbO_2_/PVDF/CC samples are shown in Figure 2a. Two characteristic taro-like peaks of the PVDF/CC substrate appear at 2*θ* = 26.0° and 43.5°, and they are well assigned to the (002) and (100) planes of carbon clothes (JCPDS no. 41-1487), respectively. The diffraction peaks of PVDF cannot be observed in the pattern of the PVDF/CC substrate; this may be assigned to the semi-crystallization characteristics of this polymer and the coated amount smaller than the resolution limit of the diffractometer. The main diffraction peaks of PbO_2_/CC and PbO_2_/PVDF/CC at 2*θ* = 25.4°, 32.0°, 36.2°, 49.1°, and 62.5° are attributable to (110), (101), (200), (211), and (301) planes of tetragonal β-PbO_2_ (JCPDS 75-2420), respectively. Notably, the peaks linked to CC disappear in the pattern of the PbO_2_/PVDF/CC composite, suggesting that the CC is compactly covered by the anchored PVDF and electrodeposited β-PbO_2_ layers, in agreement with the evidence of SEM images.

The surface chemical components and element valence states of PVDF/CC and PbO_2_/PVDF/CC composites are further revealed by XPS measurements. Their survey spectra are displayed in Figure 2b, and it can be seen that there is a particularly significant Pb 4f peak in the PbO_2_/PVDF/CC spectrum, confirming the presence of PbO_2_ particles in the outer layer of this fabricated composite. As shown in Figure 2b and Figure S3, the F 1s peak (688.15 eV) attributing to the -CH_2_-CF_2_- group of the PVDF chain can be found in the PVDF/CC spectrum and cannot be identified in the PbO_2_/PVDF/CC spectrum [34]; the F 1s peak (683.53 eV) in the PbO_2_/PVDF/CC spectrum can be assigned to residual NaF [35]. These characterization results affirm the successful synthesis of the PbO_2_/PVDF/CC composite.

**Figure 3:**
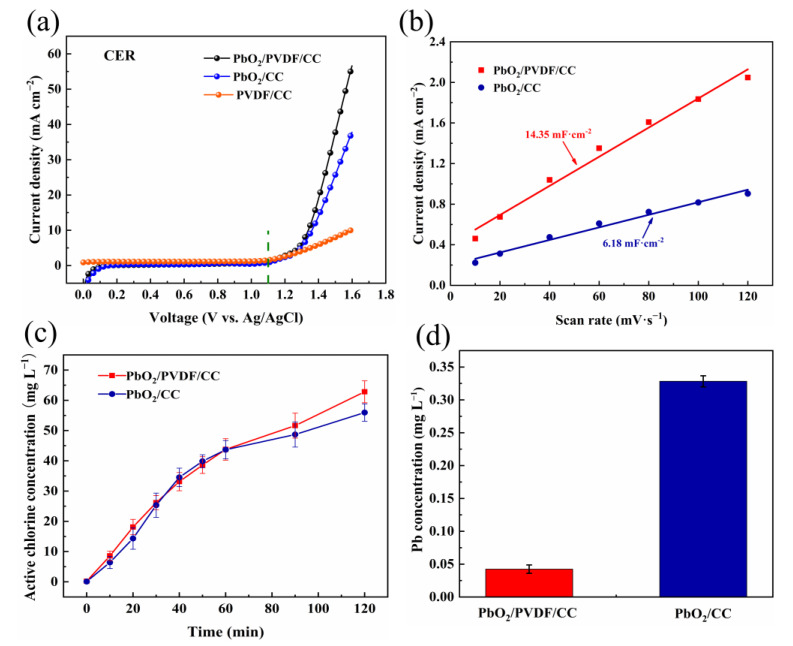
The electrochemical tests of PVDF/CC, PbO_2_/CC, and PbO_2_/PVDF/CC samples in 5 mol L^−1^ NaCl solution: (**a**) LSV polarization curves; (**b**) Plot of capacitive current against scan rate (tested potential window in a range of 0.15~0.25 V, scan rates ranging from 10 to 120 mV s^−1^); (**c**) Concentration of active chlorine; (**d**) The concentration of dissolved lead.

Figure 3c shows the core-level XPS spectrum of the Pb 4f spin–orbit doublet level. The Pb 4f spectrum of β-PbO_2_ comprises Pb (II) bands (4f _7/2_ and 4f _5/2_) centered at 137.6 eV and 142.51 eV [36], respectively, while the peaks characterizing Pb (IV) appear at 136.71 and 141.59 eV [37]. All these results verify that PbO_2_ is successfully deposited on the PVDF/CC. From the XPS analysis, the atomic ratio of Pb (IV) to Pb (II) is around 0.89 because lead dioxide polymorphs normally are not fully stoichiometric (i.e., the surface Pb from the +IV oxidation state in PbO_2_ changes moderately to +II as PbO) [38], which will result in high conductivity, thus facilitating the improvement of the PbO_2_/PVDF/CC electrode conductivity. As validated by Figure 3d, the O 1s spectral components in lattices of PbO_2_ and PbO appear at 528.7 and 529.61 eV, respectively, while those at 530.35 and 531.59 eV can be attributed to hydroxide and adsorbed oxygen species (O_ads_), respectively [31]. It is extensively accepted that the O_ads_ are related to active oxygen species (•OH). Hence, a higher proportion of O_ads_ means that more •OH free radicals can be produced [39], which will be responsible for the best performance for MO degradation. The above analysis of XPS results predicts that the prepared PbO_2_/PVDF/CC composite will have high electrical conductivity and oxidative degradation performance.

### 3.2. Electrochemical Characteristics of Fabricated Composites

The LSV measurements of PbO_2_/CC and PbO_2_/PVDF/CC composites are performed in 5 mol L^−1^ of NaCl solution and the chlorine evolution reaction (CER) properties are evaluated (Figure 3). The chlorine evolution reaction potential (CEP) value is obtained from the intersection point of the curve before and after the current density starts to increase. As shown in Figure 3a, it can be seen that the PVDF/CC has an inferior performance of chlorine evolution, and the CEPs for both PbO_2_/CC and PbO_2_/PVDF/CC composites are around 1.1 V (vs. Ag/AgCl), suggesting that the anchor of PVDF layer does not affect the chlorine evolution performance of the PbO_2_/PVDF/CC composite. As for the OER, the oxygen evolution potential (OEP) for both PbO_2_/CC and PbO_2_/PVDF/CC is about 1.25 V (vs. Ag/AgCl) (Figure S4). For the PVDF/CC electrode, similar to the CER process, this sample shows a senior OER performance (2.0 V vs. Ag/AgCl), so only the electrochemical properties of PbO_2_/CC and PbO_2_/PVDF/CC composites are involved in the following parts. With respect to the fact of CEP < OEP, we are sure the fabricated composites will exhibit an acceptable chlorine evolution selectivity in a voltage window of 0.15 V (i.e., the difference value between CEP and OEP). In addition, the electrochemical double-layer capacitance (C_dl_) and ECSA of the above two composite samples are determined by CV at different potential scan rates (Figure S5a,b) and then obtained from the plots of current density against scan rate (Figure 3b). The C_dl_ values of PbO_2_/PVDF/CC and PbO_2_/CC composites are, ca., 14.35 and 6.18 mF cm^−2^, respectively. The corresponding ECSA of PbO_2_/PVDF/CC (717.5 cm^2^) is distinctly higher than that of PbO_2_/CC (309.0 cm^2^), which will be conducive to the removal of target pollutants. As displayed by the CV curves of the PbO_2_/PVDF/CC sample (Figure S6), there is no new oxidation peak in the presence of NH_4_^+^ with a concentration of 20 mg-N L^−1^. Consequently, it can be inferred that direct electrochemical oxidization cannot be responsible for the removal of NH_4_^+^.

In order to assess the electrochemical chlorine oxidation activity, the active chlorine generation abilities of PbO_2_/PVDF/CC and PbO_2_/CC composites are investigated. Figure 3c displays the concentration variation of active chlorine along with the extension of electrolysis time. It can be directly found that the two samples can produce the identical active chlorine at the same time. Nonetheless, compared with PbO_2_/CC, we can conclude from Figure 3d that the PbO_2_/PVDF/CC composite shows a lower concentration of dissolved lead, thereby confirming that the incorporation of the PVDF layer plays a vital role in enhancing the stability and environmental safety of this fabricated composite.

### 3.3. Electrochemical Oxidation Decolorization of MO

#### 3.3.1. Effect of Conventional Parameters on Decolorization of MO

**Figure 4:**
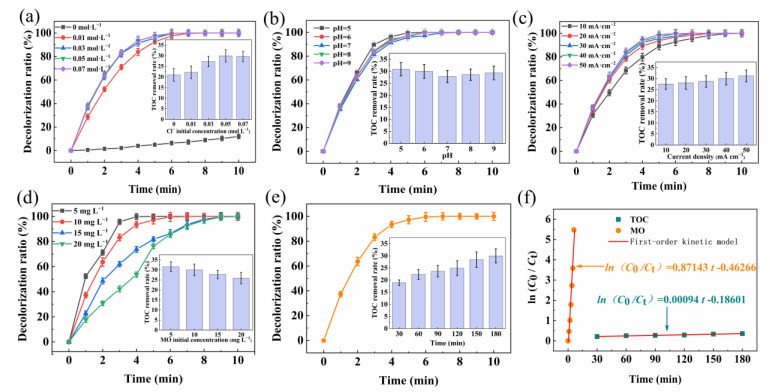
Decolorization of MO and TOC under different conditions: (**a**) Decolorization of MO and TOC under different initial Cl^−^ concentrations (current density: 40 mA cm^−2^, initial MO concentration: 10 mg L^−1^, initial pH: 6, contact time: 180 min, *T*: room temperature); (**b**) Initial pH values (current density: 40 mA cm^−2^, initial MO concentration: 10 mg L^−1^, initial Cl^–^ concentration: 0.05 mol L^−1^, contact time: 180 min, *T*: room temperature); (**c**) Decolorization of MO under different current densities (initial pH: 6, initial MO concentration: 10 mg L^−1^, initial Cl^−^ concentration: 0.05 mol L^−1^, contact time: 180 min, *T*: room temperature); (**d**) Decolorization of MO under different initial methyl orange concentrations (current density: 40 mA cm^−2^, initial Cl^−^ concentration: 0.05 mol L^−1^, initial pH: 6, contact time: 180 min, *T*: room temperature); (**e**) Decolorization rate and TOC removal rate of methyl orange under optimal conditions; (**f**) First-order kinetics of decolorization and TOC removal rate of methyl orange (initial MO concentration: 10 mg L^−1^, current density: 40 mA cm^−2^, initial Cl^−^ concentration: 0.05 mol L^−1^, initial pH: 6, contact time: 180 min, *T*: room temperature).

Batch electrolysis experiments are conducted to optimize the experimental conditions for the oxidation decolorization of MO molecules. As illustrated in Figure 4a, when Cl^–^ with an initial concentration of 0.01 M is added to the solution, a complete decolorization of MO (ca., 100%) is obtained around 10 min with the color changing from yellow to colorless, whereas, without the presence of Cl^−^, the decolorization efficiency is only 12.3%. This result suggests that, as with other reported azo dyes, the electrolysis of MO is probably chlorine-mediated and depends on Cl^−^ concentration [40]; Moreover, sodium chloride (NaCl) is used as supporting electrolyte, which also contributes to the oxidation process [41].

The decolorization ratio of MO (initial concentration of 10 mg L^−1^) with the absence of ammonium at different Cl^–^ concentrations (i.e., 0, 0.01, 0.03, 0.05, and 0.07 mol L^−1^) is investigated (Figure 4a). By increasing the initial Cl^–^ concentration from 0 to 0.07 mol L^−1^, the kinetic constant of MO decolorization rate increases from 0.0112 to 0.9098 min^−1^ (Table S1). This indicates that the increase in Cl^–^ concentration enhances the anodic oxidation of Cl^−^ to produce reactive chlorine at the anode surface, and accelerates the indirect oxidation process of MO. Thus, Cl^−^ plays the most dominant role in the decolorization of MO by the PbO_2_/PVDF/CC composite and exerts a notable effect on the decolorization efficiency of this dye. The mineralization efficiency of MO is analyzed in terms of TOC [42], and the TOC value rises with the increase in Cl^−^ concentration until 0.05 mol L^−1^; then, it almost remains invariable as the Cl^−^ concentration is higher than this value and above. When the chlorine-evolving active sites on the electrode surface are saturated, excessive Cl^−^ cannot fully contact the electrode active sites, leading to an unremarkable increase in the formation of the active chlorine and negligible improvement in MO decolorization. This can be attributed to the fact that as the concentration of chloride increases, the applied current density could become the limiting step for the chlorine-evolving reaction [43]. A similar trend is observed for the TOC removal, which reaches 29.98% and 29.69% as the added Cl^−^ concentrations are 0.05 and 0.07 mol L^−1^, respectively.

The decolorization of MO exhibits little difference at a broad pH range (pH = 5, 6, 7, 8, and 9), and a decolorization of 100% is obtained with the pH ranging from 5 to 9 after 10 min of treatment (Figure 4b), indicating the full range of pH applicability of this electrode at a concentration of 10 mg L^−1^. The reason could be attributed to the fact that the chlorine oxidation reaction occurs so fast that the pH exerts little effect on the decolorization of MO [44]. Considering the pH value of the textile dyeing and finishing wastewater (around 6~9), the pH value of 6.0 is used in the subsequent electrolysis experiments. In addition, the current density is a key parameter for controlling the reaction rate during the electrochemical oxidation process, and its effect on the decolorization of MO emerges in Figure 4c. Increasing the current density from 10 to 40 mA cm^−2^ results in the rate constant rising from 0.4461 to 0.9736 min^−1^, demonstrating that the MO decolorization has an excellent linear correlation with the applied current density. Furthermore, the decolorization of MO can be completed at 7 min when the applied current density is enhanced to 50 mA cm^−2^, being attributed to the increase in active chlorine concentration and •OH radicals [45,46]. In addition, although the increase in current density will lead to the rising risk of lead stripping from electrodes, the determined Pb^2+^ concentration is less than 0.1 mg L^−1^, consistent with the requirement of the V-level of Environmental Quality Standard for Surface Water in China (GB 3838-2002). Herein, the data of 40 mA cm^−2^ are selected as the employed current density value in this study.

The electrochemical degradation results of MO with different initial concentrations (5, 10, 15, and 20 mg L^−1^) are shown in Figure 4d. When the initial concentration increases from 5 to 20 mg L^−1^, the final decolorization rate of MO reaches over 100%, but the TOC removal rate declines from 31.59% to 25.89%, which may be due to the presence of more residual colorless intermediates of MO. Moreover, the *k* value of the pseudo-first-order kinetics decreases from 1.562 down to 0.1895 min^−1^, also suggesting the generation of a significant quantity of intermediates under conditions of high MO concentration, thus hampering the reaction between active species and MO molecules. Thus, the initial MO concentration of 10 mg L^−1^ is acceptable in this experiment. Meanwhile, when the electrolysis time reaches 180 min, MO can be rapidly decolorized in all cases and the removal of TOC is only 29.98% with the presence of 0.05 mol L^−1^ Cl^−^ (Figure 4e). For the cases of varying other parameters (Cl^−^ concentration, pH, and current density), the oxidation process of MO is still in line with the pseudo-first-order kinetics (Table S1). As displayed in Figure 4f, the rate constant of decolorization (0.87143 min^−1^) is more than two orders of magnitude larger that of TOC removal (0.00094 min^−1^). It is evident that the PbO_2_/PVDF/CC composite is admirably suitable for the decolorization of MO rather than the oxidation mineralization of this dye.

#### 3.3.2. Free Radical Scavenging Test

**Figure 5:**
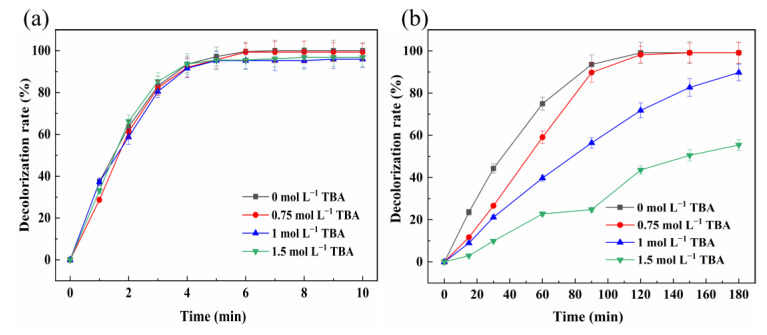
Effect of different concentrations of TBA and Cl^−^ on MO decolorization: (**a**) 0.05 mol L^−1^ Na_2_SO_4_ and 0.05 mol L^−1^ NaCl solution; (**b**) 0.05 mol L^−1^ Na_2_SO_4_ (initial MO concentration: 10 mg L^−1^, current density: 40 mA cm^−2^, initial Cl^−^ concentration: 0.05 M, initial pH: 6, contact time: 180 min, *T*: room temperature).

The reactive oxygen species masking experiment is employed to identify the predominant free radical species in the oxidation process of the PbO_2_/PVDF/CC composite. TBA can effectively quench •OH [47,48,49], and the addition of TBA significantly imposes a noticeable restriction on the removal of MO. As shown in Figure 5a, with the TBA concentration increasing from 0 to 1.5 mol L^−1^, the decolorization rate of MO decreases gradually. More importantly, when the concentration of TBA is enhanced to 1.5 mol L^−1^, the suppression effect is more apparent, confirming that •OH plays an important role in the reaction. After 10 min of reaction, there is a slight decrease in decolorization rate (from 100% to 96.8%) with the further increase in TBA concentration. It could be concluded that excessive TBA restricts the MO decolorization of •OH, but the in situ electrogenerated active chlorine is responsible for the MO decolorization. In addition, the scavenging test in the absence of Cl^–^ is tested in Figure 5b, and the suppression effect becomes more obvious with the presence of 1.5 mol L^−1^ of TBA; in this case, the decolorization rate of MO is only 55.45% after electrolysis for 180 min. Based on these results, it can be concluded that the decolorization of MO by the PbO_2_/PVDF/CC composite takes place via the oxidation process of active chlorine. Of course, the •OH also contributes to the oxidation decolorization of this dye.

### 3.4. Electrochemical Degradation of MO with the Presence of Ammonium

**Figure 6:**
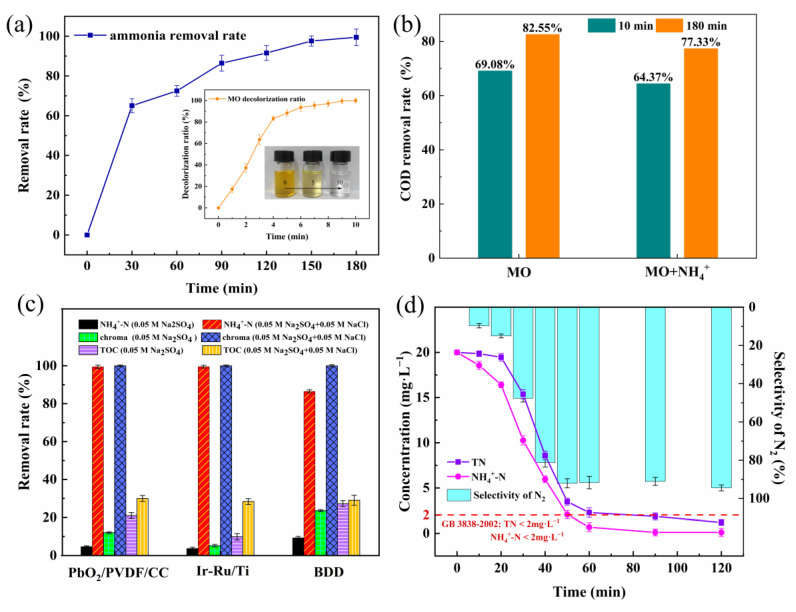
Removal of MO with the presence of ammonium: (**a**) MO and NH_4_^+^ removal rate (initial NH_4_^+^-N concentration: 20 mg L^−1^, initial MO concentration: 10 mg L^−1^, current density: 40 mA cm^−2^, initial Cl^−^ concentration: 0.05 mol L^−1^, initial pH: 6, contact time: 180 min, *T*: room temperature); (**b**) COD removal rate of MO and MO + NH_4_^+^ systems (initial NH_4_^+^-N concentration: 20 mg L^−1^, initial MO concentration: 10 mg L^−1^, current density: 40 mA cm^−2^, initial Cl^−^ concentration: 0.05 mol L^−1^, initial pH: 6, contact time: 180 min, *T*: room temperature); (**c**) Removal rate of NH_4_^+^-N, chroma, and TOC with different oxidation systems (initial NH_4_^+^-N concentration: 20 mg L^−1^, initial MO concentration: 10 mg L^−1^, current density: 40 mA cm^−2^, initial Cl^−^ concentration: 0.05 mol L^−1^, initial pH: 6, contact time: 180 min, *T*: room temperature); (**d**) The concentrations of TN, NH_4_^+^-N, and the selectivity of N_2_ (initial NH_4_^+^-N concentration: 20 mg L^−1^, current density: 40 mA cm^−2^, initial Cl^−^ concentration: 0.05 mol L^−1^, initial pH: 6, contact time: 120 min, *T*: room temperature).

As depicted in Figure 6a, the removal rate of ammonium increases and the chroma of MO decays gradually as the reaction progresses. In addition, a favorable removal rate of NH_4_^+^ (99.43%) after 180 min and a superb decolorization rate of MO (100%) within 10 min can be obtained. The presence of ammonium shows an inconspicuous effect on the MO decolorization, suggesting that MO and NH_4_^+^ can be removed synchronously using the PbO_2_/PVDF/CC composite. Compared with that of the individual MO system, it can be observed from Figure 6b that the COD removal of the MO and NH_4_^+^ coexisting system (MO + NH_4_^+^) slightly decreases from 82.55% to 77.33%; after treatment for 180 min, the COD values for these two systems are less than 40 mg L^−1^ (initial COD value: MO system: 126.92 mg L^−1^, MO + NH_4_^+^: 117.23 mg L^−1^), meeting the requirement of the V-level of Chinese Surface water Quality. The influence of indirect chlorine oxidation on the decolorization of MO is further validated by the comparison of those of boron-doped diamond electrodes (BDDs) and commercial chlorination electrodes (Ir-Ru/Ti) in Figure 6c. All three electrodes show an excellent effect on MO decolorization (100%). Strikingly, the PbO_2_/PVDF/CC obtains a superb NH_4_^+^ removal of 99.48%, bearing a close resemblance to the Ir-Ru/Ti electrode (~100%). As Cl^–^ coexists with a concentration of 0.05 mol L^−1^, PbO_2_/PVDF/CC and Ir-Ru/Ti show similar removal efficiencies, while BDD exhibits a slightly low efficiency due to the insufficient electrogeneration of active chlorine. Thus, we are sure that the electrochemical oxidation process with the presence of Cl^−^ deserves to be adopted.

With and without the presence of NH_4_^+^, there is no obvious difference in TOC removal (varying from 29.76% to 29.98%) after a reaction of 180 min, and the three parameters (decolorization rate, COD removal rate, and TOC removal rate) follow the order of decolorization rate > COD removal rate > TOC removal rate, demonstrating the fact that •OH and active choline species show the robust efficiencies for destroying the azo bond of the chromophore (decolorization process), rather than the ring opening and bond breaking process of MO (COD removal) as well as the mineralization process of this dye (TOC removal) [50].

The experimental results of the chlorine oxidation of NH_4_^+^ alone by the PbO_2_/PVDF/CC composite under different experimental conditions are depicted in Table S2, and a favorable removal rate (99.45%) for NH_4_^+^ can be obtained in 180 min (Figure S7). Strikingly, the concentration of leached Pb is lower than 0.1 mg L^−1^, attesting to the acceptable stability of the fabricated composite. It should be mentioned that the oxidation efficiency of NH_4_^+^ in the presence of 0.05 mol L^−1^ of Cl^−^ (99.48%) is significantly higher than that of the case of nonexistent Cl^−^ (4.663%), showing that the indirect electrochemical chlorine oxidation dominates the oxidation removal of this nitrogen-based pollutant. In addition, to explore the transformation pathway and nitrogen-based intermediates during the process of chlorine oxidation, the percentage of nitrogen species is monitored (Figure S8), and the concentrations of TN and NH_4_^+^-N (Figure 6d) progressively reduce during the reaction process and reaches 0.103 mg L^−1^ and 1.204 mg L^−1^, respectively, complying with the threshold limit of the Chinese V-level of surface water environmental quality (2 mg L^−1^). The N_2_ selectivity is also as high as 94.46%. It can be attested that the PbO_2_/PVDF/CC composite exhibits a charming efficiency for electrochemical conversion of NH_4_^+^ to N_2_.

Based on the outlined facts mentioned above, this work can provide the strategy for the removal of dye pollutants and the electrochemical denitrification of ammonium-based eutrophication pollution.

### 3.5. Electrochemical Conversion Pathways of MO and Ammonium

The oxidation decolorization process of MO is analyzed by the UV-Visible spectrophotometer, as shown by Figure S9. The broad absorption band at 465 nm for the characteristic reflection peak linked to the N=N chromogenic group of MO gradually disappears after 10 min of treatment, suggesting the rapid N=N break of MO molecules. The enhancement in absorption for the peak of 250 nm means the formation of some by-products during the oxidation process of MO. As the reaction time is prolonged, MO molecules are further degraded and mineralized, verified by the weakness of the peak strength and the decreases in COD and TOC reported in Figure 6.

**Figure 7:**
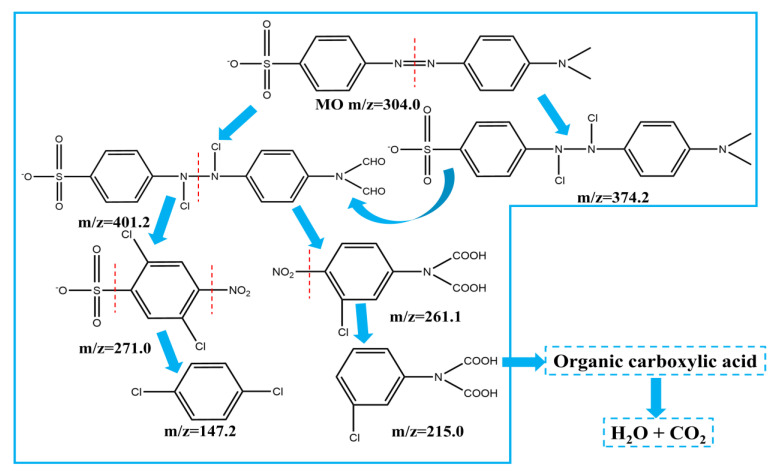
Degradation pathway of MO via the electrochemical oxidation of PbO_2_/PVDF/CC composite.

To clarify the degradation pathway of MO in the electrochemical oxidation process, the generated intermediates are identified by HPLC-MS, and the mass spectra of intermediates are displayed in Figure S10; after the beginning of the reaction, the fragmentation pattern of the MO molecule (*m*/*z* = 304.0) cannot be discerned, indicating the occurrence of the oxidation reaction. The MS fragmentation patterns corresponding to different intermediates are given in Figure S10b–e, and the proposed degradation pathway of MO via the electrochemical oxidation process of the PbO_2_/PVDF/CC composite is illustrated in Figure 7.

The major pathway involves the bond break of the azo group (-N=N-) by the attacks of active species (•OH and active chlorine) [51], and the -N-Cl bond is formed. In addition, methyl groups are oxidized to aldehydes or carboxylic acids [28]. As the oxidation reaction proceeds, the N=N bond is cleaved completely and the MO molecule is converted to different types of nitrobenzene compounds [52]. For intermediates of benzene compounds, the benzene ring can be hydrogenated by •OH, and reactive chlorine is available to form different intermediates [53]. Finally, with the further oxidation processes of active species, chlorine-based substituents and other organic intermediates can eventually convert to CO_2_, H_2_O, and the corresponding anions (Cl^−^, NO_3_^−^, and SO_4_^2−^) [28,54].

For the oxidation removal of ammonium by the PbO_2_/PVDF/CC composite, Cl^–^ is converted to Cl_2_ on the composite surface (Equation (1)), then the formed Cl_2_ dissolves in solution to produce HClO (Equation (2)). The pollutant of ammonium is mainly oxidized to nitrogen gas by HClO, and the reaction process is as follows in Equation (3); the detailed illustration of this process is presented in the Supporting Information. Based on above interpretations, the conversion pathways of ammonium via an electrochemical oxidation process on the surface of the PbO_2_/PVDF/CC composite is briefly displayed in Figure S11.

2Cl^−^ − 2e^−^ → Cl_2_ (1)

Cl_2_ + H_2_O ↔ HClO + HCl (2)

3HClO + 2NH_4_^+^ → N_2_^↑^ +3Cl^−^ + 5H^+^ + 3H_2_O (3)

### 3.6. Stability of PbO_2_/PVDF/CC Composite

**Figure 8:**
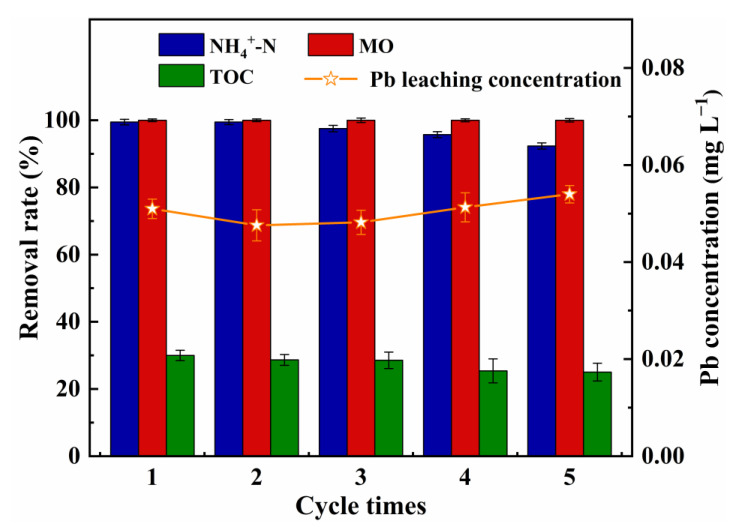
The stability test of the PbO_2_/PVDF/CC composite in terms of removals of MO, NH_4_^+^-N, and TOC as well as the leaching concentration of Pb (initial NH_4_^+^-N concentration: 20 mg L^−1^; initial MO concentration: 10 mg L^−1^; current density: 40 mA cm^−2^; initial Cl^−^ concentration: 0.05 mol L^−1^; initial pH: 6; contact time: 120 min; *T*: room temperature).

Generally, the stability and environmental friendliness of the PbO_2_/PVDF/CC composite will be of great importance with respect to the engineering perspective. Thus, the endurance test of this composite is conducted and the results are presented in Figure 8. After five runs, the electrochemical oxidation efficiencies of MO and NH_4_^+^ remain at high levels (~100% of decolonization rate for MO, removal rate > 92% for NH_4_^+^). In addition, the TOC removal rate decreases mildly from 29.98% to 25.03%, and the leached concentration of Pb (0.054 mg L^−1^) is still lower than the limited 0.1 mg L^−1^, hence corroborating the long-term stability of this composite.

**Figure 9:**
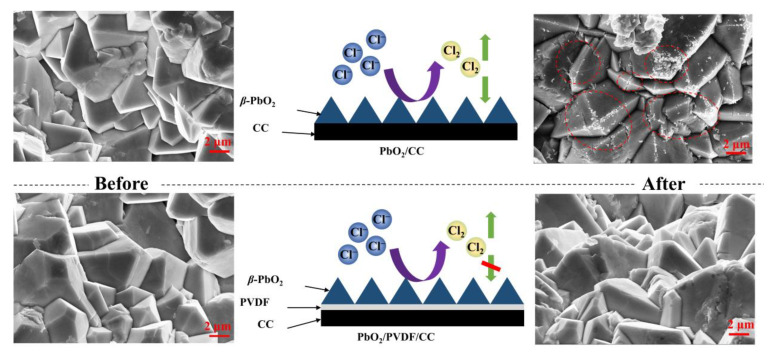
Surface morphologies of PbO_2_/CC PbO_2_/PVDF/CC before and after five−cycle test.

The SEM images (Figure 9) show the changes in surface morphologies of PbO_2_/CC and PbO_2_/PVDF/CC composites over five cycles. Before the test, both show typical pyramid-like structures, with the presence of smooth surfaces and without obvious cracks. After the treatment of the electrochemical oxidation process, the morphology of the PbO_2_/CC sample is quite different from that of the PbO_2_/PVDF/CC composite, and there are many detected cracks on the surface of PbO_2_/CC (shown in the red circle in Figure 9), which may be due to the impacting effect of generated microbubbles (Cl_2_ and some of O_2_) diffusing between the PbO_2_ active layer and the CC matrix; of course, the erosion destruction of generated Cl_2_ microbubbles should also be involved. By contrast, there are no obvious cracks on the surface of the PbO_2_/PVDF/CC composite, and its surface morphology remains a complete pyramid aspect due to the role of the PVDF layer as an oxidation-resistance buffer between the active layer and the CC matrix. The PVDF buffer layer can efficiently prevent Cl_2_ microbubbles from corroding the CC matrix and reduce the lead dissolution and volume expansion of this composite, thereby guaranteeing the stability and environmental safety of the PbO_2_/PVDF/CC composite.

## 4. Conclusions

In this paper, a PbO_2_/PVDF/CC composite was fabricated via a facile surface coating and galvanostatic electrodeposition process, which contributes insight into enhancing the stability of the PbO_2_-based composite. This fabricated composite was employed for electrochemical oxidation removals of methyl orange and ammonium in aqueous solution, and the properties of oxidation efficiency and durability for this composite were evaluated. The following core results are obtained.

(1) The fabricated PbO_2_/PVDF/CC composite shows a low chlorine evolution potential (1.1 V vs. Ag/AgCl) and a high oxygen evolution potential (1.25 V vs. Ag/AgCl), which is helpful to facilitate the process of chlorine electrochemical oxidation.
(2) The PbO_2_/PVDF/CC composite shows a strong decolorization efficiency of methyl orange (~100%), excellent removal of ammonium (>99%), and minimal concentration of lead leaching (0.054 mg L^−1^), suggesting its predicted engineering application.
(3) In addition, the fabricated PbO_2_/PVDF/CC composite exhibits desirable stability and environmental safety. After five runs, based on the potential application prospect, the decolorization efficiency of methyl orange, the removal rate of ammonium, and the leached concentration of Pb remain at acceptable levels. The PbO_2_/PVDF/CC composite deserves to be exploited for wastewater treatment in the textile dyeing and finishing industry.

## Data Availability

Data will be made available on request.

## Supplementary Materials

The following supporting information can be downloaded at: https://www.mdpi.com/article/10.3390/polym15061462/s1, Figure S1: SEM images of samples; Figure S2: SEM images of PbO_2_/PVDF/CC composite with different electrodeposition time; Figure S3: F 1s core level spectra for PbO_2_/PVDF/CC and PbO_2_/PVDF/CC composites; Figure S4: Linear sweep voltammetry polarization curves of tested samples in 0.5 mol L^−1^ H_2_SO_4_ solution; Figure S5: The successive CV curves; Figure S6: Cyclic voltammogram of PbO_2_/PVDF/CC composite without and with 20 mg L^−1^ NH_4_^+^-N in 0.05 mol L^−1^ Na_2_SO_4_ and 0.05 mol L^−1^ NaCl solution; Figure S7: Obtained results of NH_4_^+^ oxidation via the PbO_2_/PVDF/CC composite anode; Figure S8: Percentage of obtained product during the electrochemical process of NH_4_^+^; Figure S9: UV-Vis results for the analysis of MO decolorization; Figure S10: Determined MS results for MO electrochemical oxidation by the employment of PbO_2_/PVDF/CC composite; Figure S11: The proposed conversion pathway of ammonium; Table S1: The results of electrochemical chlorine oxidation for MO; **Table S2**: The results of electrochemical chlorine oxidation for ammonium. References [55,56] are cited in the Supplementary Materials.
