# Supplementary material for: Fabrication of PbO2/PVDF/CC Composite and Employment for the Removal of Methyl Orange
Source: Polymers (Basel). 2023 Mar 15;15(6):1462. doi: 10.3390/polym15061462 (PMC10053905; doi:10.3390/polym15061462)
Supplement: Supplementary file 1 [file polymers-15-01462-s001.zip › polymers-2265890-supplementary.pdf]

Supplementary Material
Fabrication of PbO_2/PVDF/CC composite and the employment for removal of methyl orange

Laizhou Song*[1], Cuicui Liu[1], Lifen Liang[2], Yalong Ma[1], Xiuli Wang[1], Jizhong Ma[1], Zeya Li[1], Shuqin Yang[1]

[1] Hebei Key Laboratory of Heavy Metal Deep-Remediation in Water and Resource Reuse, School of Environmental and Chemical Engineering, Yanshan University, Qinhuangdao 066004, China.
[2] Hebei University of Environmental Engineering, Qinhuangdao 066102, Hebei, China
* Correspondence: songlz@ysu.edu.cn, Tel.: +86-335-8387741; Fax: +86-335-8061569.

S1. Materials

Acetylene black (AB), calcium carbonate ($CaCO_3$), N-methyl-2-pyrrolidinone (NMP), sulfuric acid (H_2SO_4, 98 wt%), lead nitrate ($Pb(NO_3)_2$), nitric acid (HNO_3, 68 wt%), ammonium sulfate ($(NH_4)_2SO_4$), sodium fluoride (NaF), potassium dichromate ($K_2Cr_2O_7$), silver sulfate (Ag_2SO_4), ammonium ferrous sulfate hexahydrate ($Fe(NH_4)_2(SO_4)_2{\cdot}6H_2O$), methyl orange (MO), sodium chloride (NaCl), anhydrous sodium sulfate (Na_2SO_4), sodium hydroxide (NaOH), ferrous sulfate heptahydrate ($FeSO_4{\cdot}7H_2O$), hydrogen peroxide (H_2O_2, 30 wt%), tertiary butanol (TBA, 99.5 wt%), acetone and absolute ethanol were purchased from Tianjin Kemiou Chemical Reagent Co. Ltd. (Tianjin, China). All the above analytical reagents were used as received and without further purification. The carbon cloth (CC) was provided by Yu Bo Biotech Co. Ltd. (Shanghai, China). The employed polyvinylidene fluoride (PVDF) polymer was the lab-based waste PVDF membrane; before usage, it was washed using ethanol and deionized water and then dried in a drying oven at 60℃ overnight. In addition, deionized water with a conductivity value < 1.0 μS/cm for the preparation of all solutions was produced by a ULUPURE purification system (UPR-I-60L, Sichuan, China).

S2. Pretreatment of carbon cloth

To remove greasy dirties or impurities on the surface of CC, prior to the use, the CC sample (2.0 cm × 2.0 cm) was sequentially immersed in an absolute ethanol solution, acetone (room temperature for 6 h) and then treated by sonication-assisted washing with deionized water. After that, the CC sample was dried in an oven at 65 ºC overnight.

S3. Calculations of electrochemical surface area (ECSA)and efficiencies

S3.1. Calculations of electrochemical surface area (ECSA)

The double-layer capacitance (C_{dl}) was applied to calculate the electrochemical active surface areas (ECSA) [55]. The C_{dl} values of the electrodes were calculated by cyclic voltammetry (CV) between 0.15 and 0.25 V (vs. Ag/AgCl) at the scan rate in the range of 10-120 mV s^{-1}. The calculation details of the double-layer capacitance (C_{dl}) values and the electrochemical active surface areas (ECSA) are calculated by the following equations:

$$C_{dl} = \frac{J}{V} \tag{S1}$$

$$ECSA = \frac{C_{dl}}{C_s} \times S \tag{S2}$$

where $J = (j_+ - j_-)/2$, j_+ and j_- are the current density at 0.2 V (vs Ag/AgCl) in the CV curves, V the scan rate (mV s^{-1}), C_{dl} the double layer capacitance, C_s the general specific capacitance (generally in a certain range of 0.02-0.06 mF cm^{-2}, S the geometric area of the working electrode, and 0.02 mF cm^{-2} is adopted in this study) [55].

S3.2. Calculation of the N_2 selectivity (S_{N2})

Assuming that N_2 is the main product of ammonium chlorine oxidation, the generated amount of nitrogen gas is obtained from the mass balance of the total nitrogen content Equation S3. The N_2 selectivity (S_{N2}) is defined as conforming to Equation S4.

$$[N_2] = [NH_4^+] - [NO_3^-] - [NO_2^-] - [NH_2Cl] - [NHCl_2] - [NCl_3] \quad \text{(S3)}$$

$$S_{N_2}(\%) = \frac{[N_2]_t}{[NH_4^+]_0 - [NH_4^+]_t} \times 100\% \quad \text{(S4)}$$

where all the elements were calculated as N, S_{N2} represents the selectivity of N_2, $[N_2]_t$ is N_2 generation concentration, $[NH_4^+]_0$ and $[NH_4^+]_t$ are the concentration of ammonium (mg-N L^{-1}) at initial and time *t*.

S3.3. Calculations of current efficiency

The current efficiency (CE) is a key parameter for evaluating practical efficiency [56] and it can be calculated as Equation S5.

$$CE_i(\%) = \frac{\Delta C_{NH_4^+} \times n_i \times F \times V}{M \times \int_0^t I dt} \times 100 \quad \text{(S5)}$$

where *F* represents the Faraday constant (96485 C mol^{-1}), $\Delta C_{NH_4^+}$ the concentration variation of NH_4^+ (mg-N L^{-1}), n_i the number of electrons involved in the generation of NO_3^- (n = 8) or N_2 (n = 3), *V* the volume of the electrolyte (L), *M* the molar mass of nitrogen (14000 mg mol L^{-1}), *I* the applied current intensity (A cm^{-2}), and *t* is the electrolysis time (s).

S3.4. Calculations of removal efficiencies

The NH_4^+ removal efficiency ($R_{NH_4^+}$), decolorization efficiency (R_{color}), TOC removal (R_{TOC}), COD removal (R_{COD}) and total nitrogen concentration (*TN*) are calculated according to the following equations.

$$R_{NH_4^+}(\%) = \frac{[NH_4^+]_0 - [NH_4^+]_t}{[NH_4^+]_0} \times 100 \quad \text{(S6)}$$

$$R_{COD}(\%) = \frac{[COD]_0 - [COD]_t}{[COD]_0} \times 100 \quad \text{(S7)}$$

$$R_{color}(\%) = \frac{A_0 - A_t}{A_0} \times 100 \quad \text{(S8)}$$

$$R_{TOC}(\%) = \frac{[TOC]_0 - [TOC]_t}{[TOC]_0} \times 100 \quad \text{(S9)}$$

$$[TN] = [NH_4^+]_0 - [N_2]_t \quad \text{(S10)}$$

Where $[NH_4^+]_0$, $[COD]_0$, $[TOC]_0$, are the initial concentrations of NH_4^+, COD and TOC, separately; $[NH_4^+]_t$, $[COD]_t$, $[TOC]_t$, $[N_2]_t$ represent their concentrations at time *t*. A_0 and A_t are the absorbance of MO at the initial and the given time *t*, respectively.

S4. Supporting Figures

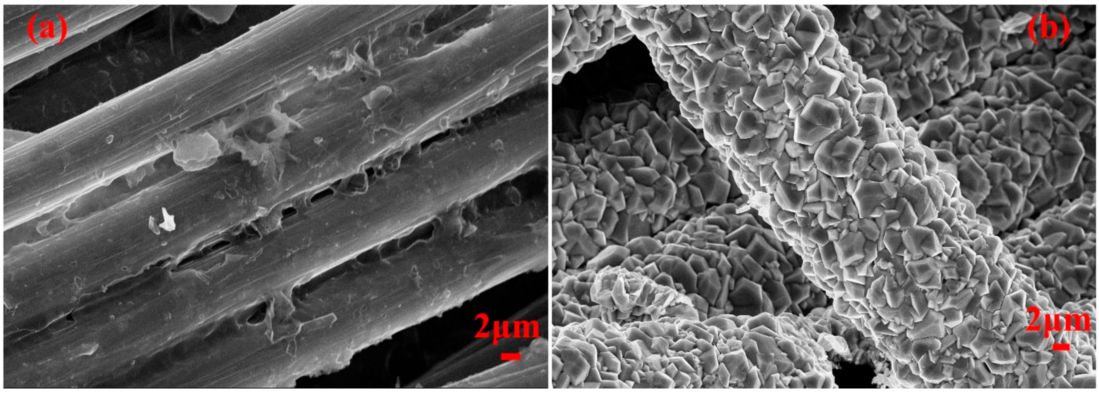

Figure S1. SEM images of samples: (**a**) Without addition $CaCO_3$; (**b**) SEM images of PbO_2/CC composite.

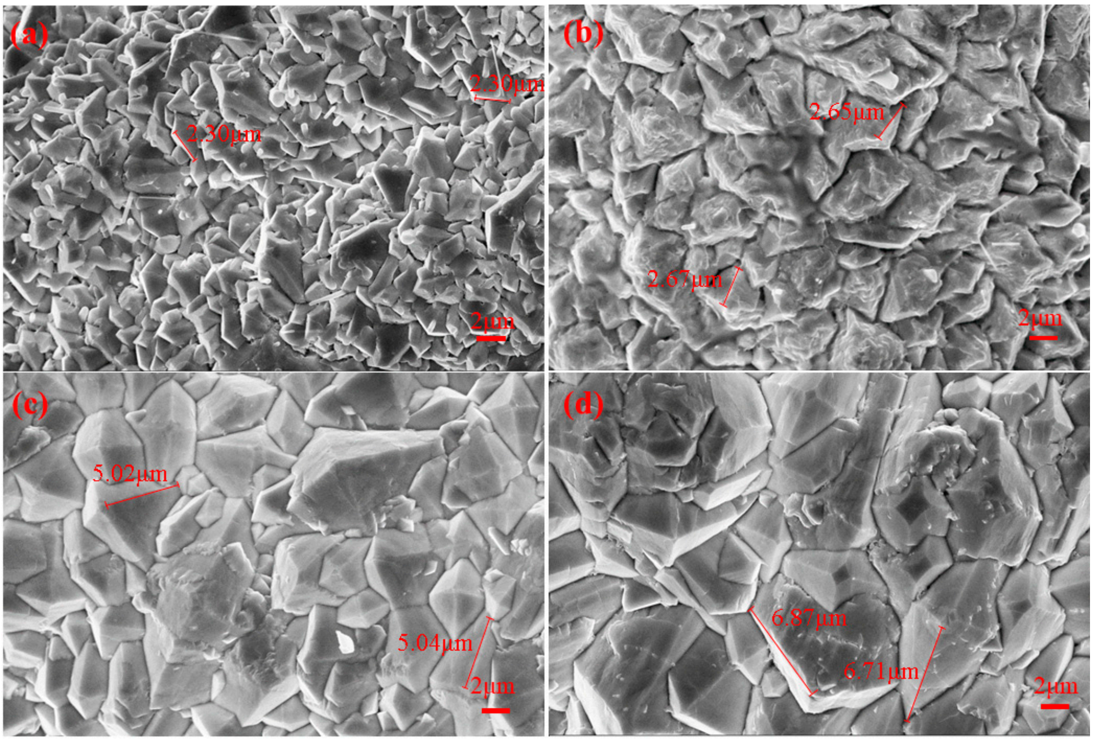

Figure S2. SEM images of PbO_2/PVDF/CC composite with different electrodeposition time: (**a**) 5 min; (**b**) 10 min; (**c**) 20 min and (**d**) 25 min, respectively.

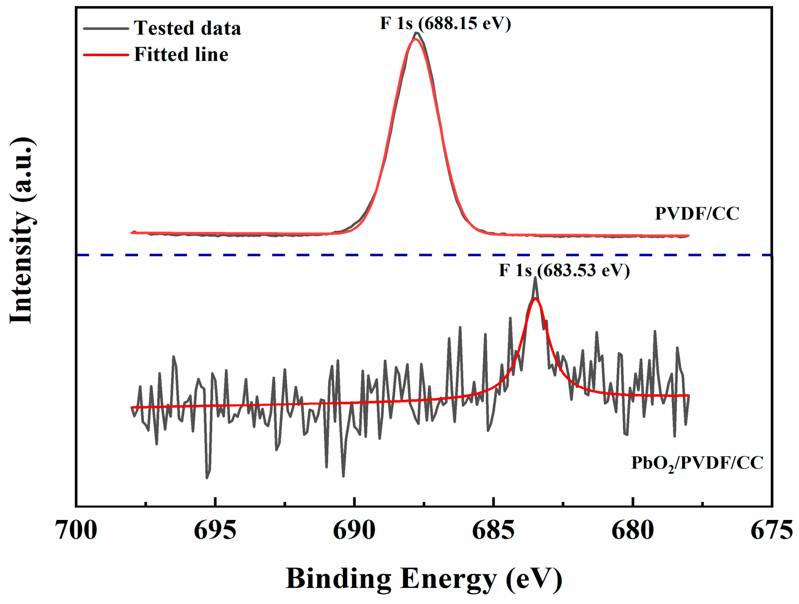

Figure S3. F 1s core level spectra for PbO_2/PVDF/CC and PbO_2/PVDF/CC composites.

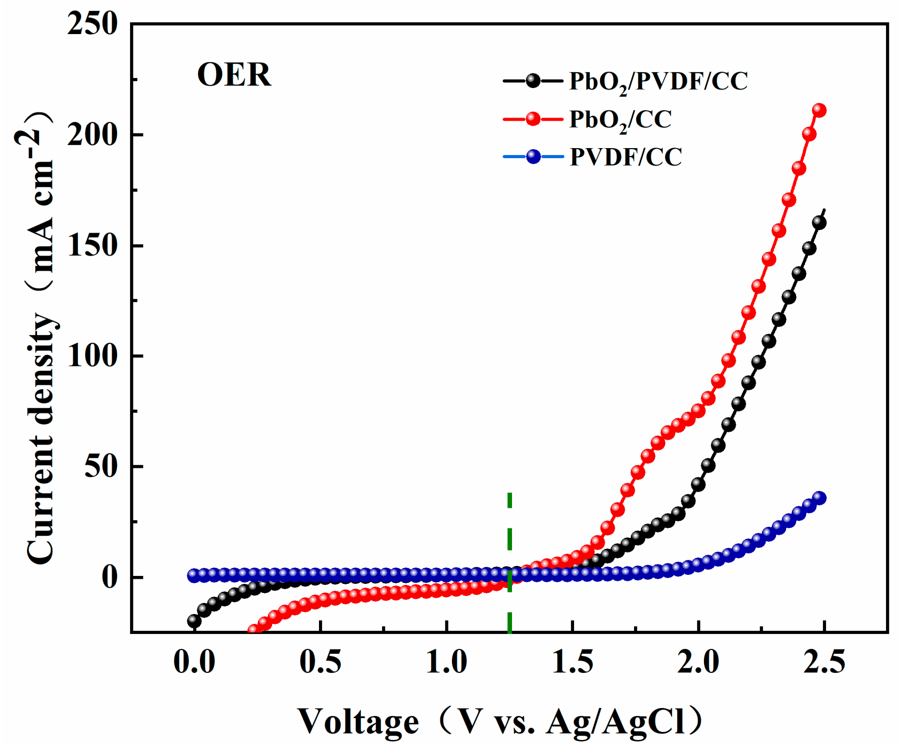

Figure S4. Linear sweep voltammetry polarization curves of tested samples in 0.5 mol L^{-1} H_2SO_4 solution.

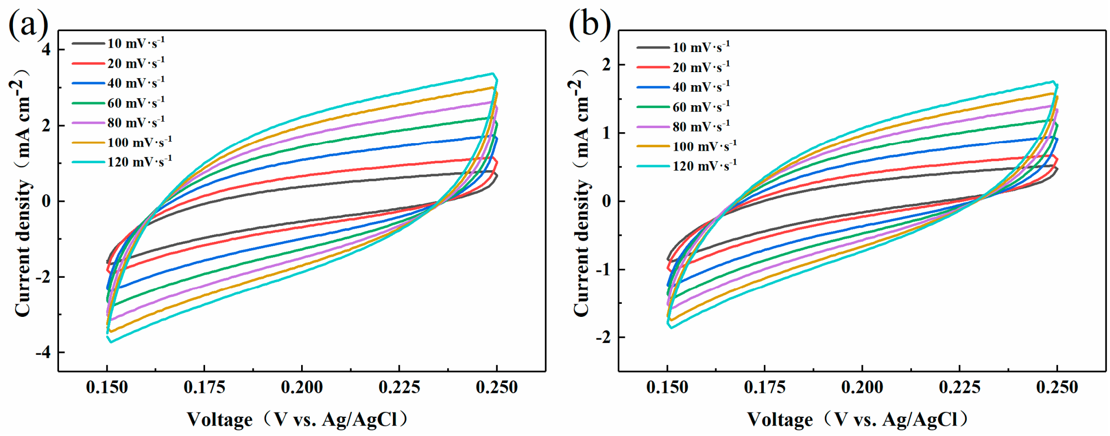

Figure S5. The successive CV curves: (**a**) PbO_2/PVDF/CC and (**b**) PbO_2/CC tested between 0.15 and 0.25 V at scan rates from 10 to 120 mV s^{-1} in 5 mol L^{-1} NaCl solution.

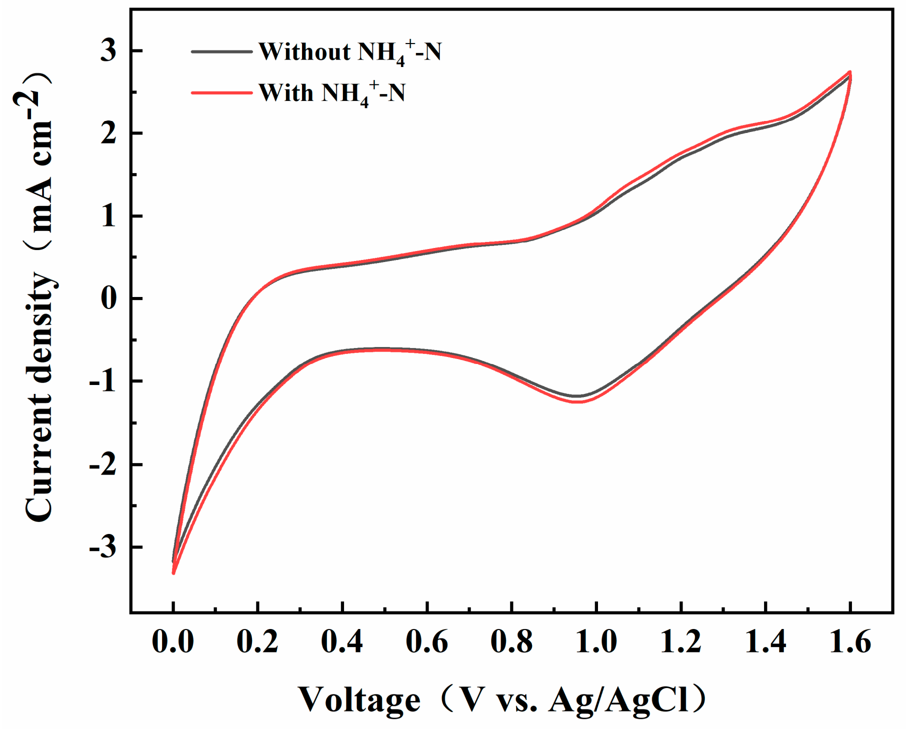

Figure S6. Cyclic voltammogram of PbO_2/PVDF/CC composite without and with 20 mg L^{-1} NH_4^+-N in 0.05 mol L^{-1} Na_2SO_4 and 0.05 mol L^{-1} NaCl solution.

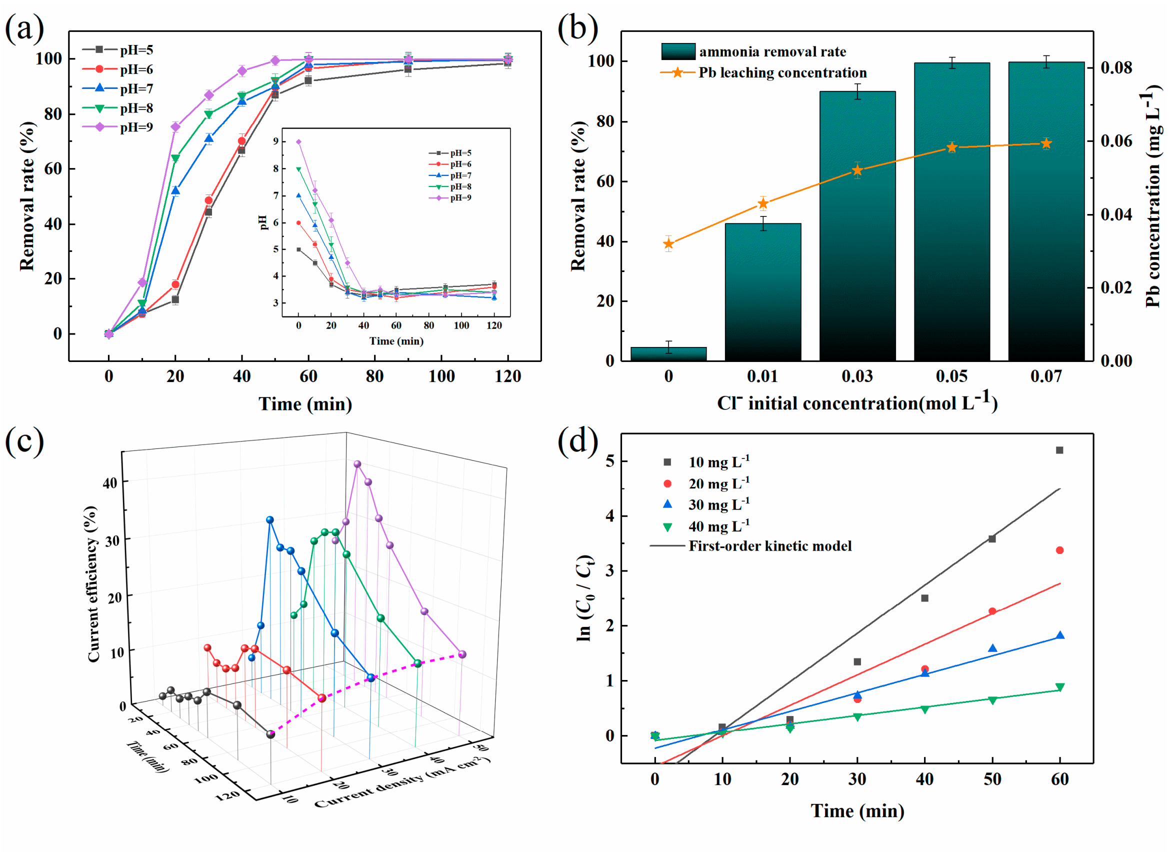

Figure S7. Obtained results of NH_4^+ oxidation via the PbO_2/PVDF/CC composite anode: (**a**) Effect of pH values (current density: 40 mA cm^{-2}, initial NH_4^+-N concentration: 20 mg L^{-1}, initial Cl^- concentration: 0.05 mol L^{-1}, contact time: 120 min, *T*: room temperature); (**b**) NH_4^+ removal rate and lead leached concentration under different initial Cl^- concentration (initial pH: 6, current density: 40 mA cm^{-2}, initial NH_4^+-N concentration: 20 mg L^{-1}, contact time: 120 min, *T*: room temperature); (**c**) Current efficiency of NH_4^+ oxidation at different current density (initial pH: 6, initial NH_4^+-N concentration: 20 mg L^{-1}, initial Cl^- concentration: 0.05 mol L^{-1}, contact time: 120 min, *T*: room temperature); (**d**) Curves of first-order kinetic model for NH_4^+ oxidation with different initial ammonium concentration (initial pH: 6, current density: 40 mA cm^{-2}, initial Cl^- concentration: 0.05 mol L^{-1}, contact time: 120 min, *T*: room temperature).

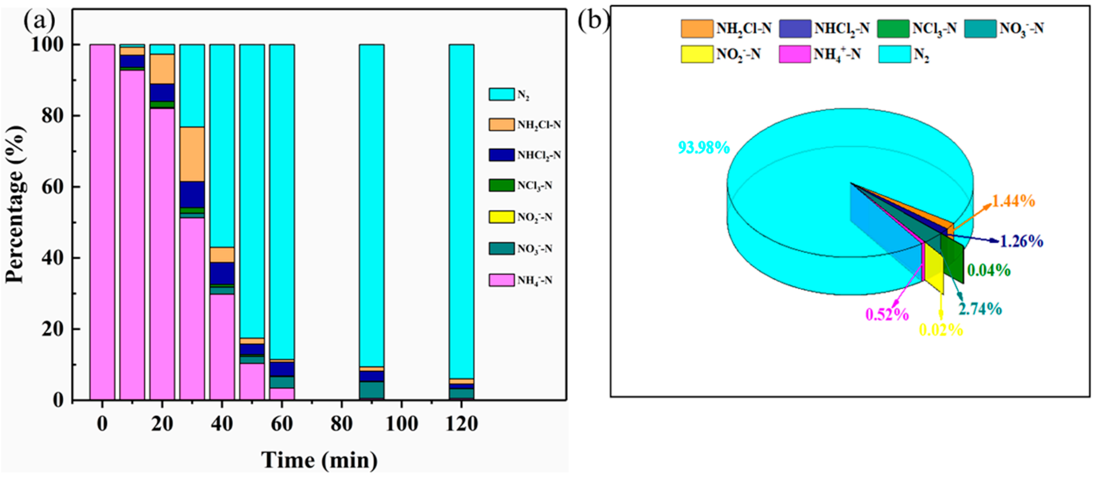

Figure S8. Percentage of obtained product during the electrochemical process of NH_4^+: (**a**) Distribution of nitrogen species with time; (**b**) The proportion of nitrogen species (current density: 40 mA cm^{-2}, initial NH_4^+-N concentration: 20 mg L^{-1}, initial Cl^- concentration: 0.05 mol L^{-1}, initial pH: 6, contact time: 120 min, *T*: room temperature).

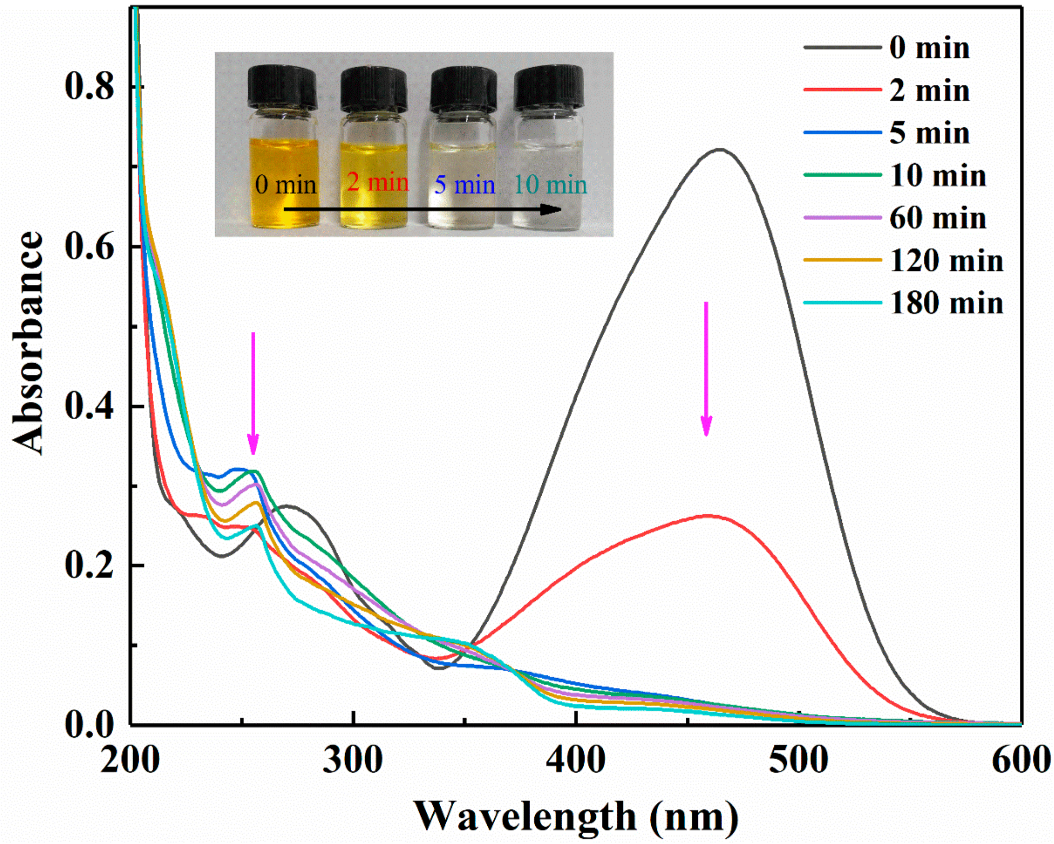

Figure S9. UV-Vis results for the analysis of MO decolorization.

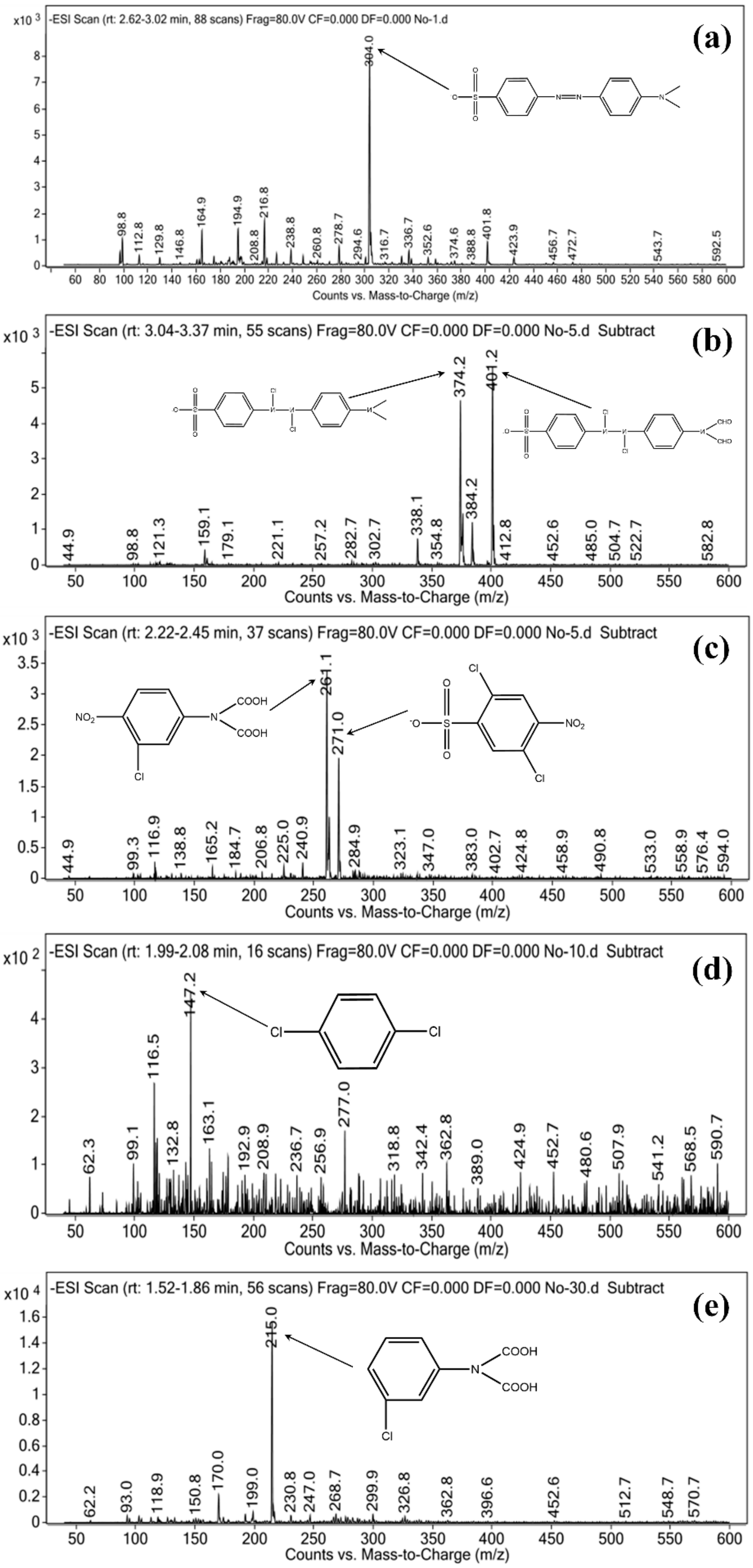

Figure S10. Determined MS results for MO electrochemical oxidation by the employment of PbO_2/PVDF/CC composite.

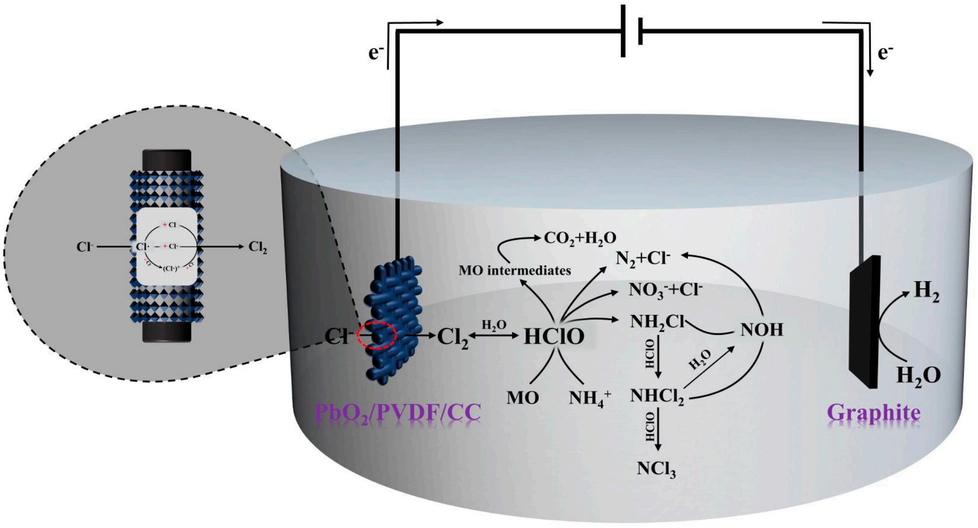

Figure S11. The proposed conversion pathway of ammonium.

S5. Supporting Tables

Table S1. The results of electrochemical chlorine oxidation for MO.

Initial pH	Initial Cl^- ($mol·L^{-1}$)	Current Density ($mA·cm^{-2}$)	Initial MO ($mg·L^{-1}$)	MO-Colour k (min^{-1})	MO-Colour R^2	MO-Colour 10 min removal rate (%)	MO-TOC k ($×10^{-4}$ min^{-1})	MO-TOC R^2	MO-TOC 180 min removal rate (%)	Pb Leaching concentration ($mg·L^{-1}$)
5	0.05	40	10	1.1689	0.9522	100	10.38	0.9710	30.85	0.0724
6	0.05	40	10	0.8714	0.9500	100	9.449	0.9765	29.98	0.0533
7	0.05	40	10	0.6329	0.9921	100	8.643	0.9792	27.85	0.0501
8	0.05	40	10	0.9868	0.8756	100	9.109	0.9827	28.59	0.0325
9	0.05	40	10	0.8913	0.9373	100	8.710	0.9463	29.32	0.0345
6	0	40	10	0.0112	0.9744	12.03	13.18	0.9804	21.01	0.0215
6	0.01	40	10	0.5986	0.9577	100	13.32	0.9578	22.19	0.0412
6	0.03	40	10	0.8313	0.9174	100	7.057	0.9890	27.27	0.0487
6	0.05	40	10	0.8714	0.9500	100	9.449	0.9765	29.98	0.0533
6	0.07	40	10	0.9098	0.9407	100	9.479	0.9895	29.69	0.0594
6	0.05	10	10	0.4461	0.9917	100	9.228	0.9680	27.54	0.0287
6	0.05	20	10	0.5830	0.9955	100	8.891	0.9744	28.02	0.0265
6	0.05	30	10	0.6509	0.9935	100	8.959	0.9732	28.74	0.0415
6	0.05	40	10	0.8714	0.9500	100	9.449	0.9765	29.98	0.0533
6	0.05	50	10	0.9736	0.9578	100	10.56	0.9796	31.21	0.0574
6	0.05	40	5	1.562	0.8678	100	9.669	0.9894	31.59	0.0487
6	0.05	40	10	0.8714	0.9500	100	9.449	0.9765	29.98	0.0533
6	0.05	40	15	0.3381	0.9962	100	9.988	0.9952	27.68	0.0585
6	0.05	40	20	0.1895	0.9983	100	9.324	0.9456	25.89	0.0578

Table S2. The results of electrochemical chlorine oxidation for ammonium.

Initial pH	Initial Cl^- (mol L^{-1})	Current density (mA cm^{-2})	Initial NH_4^+-N (mg L^{-1})	k ($×10^{-3}$ min^{-1})	R^2	NH_4^+-N removal rate (%)	Pb leaching concentration (mg L^{-1})
5	0.05	40	20	44.47	0.9025	98.43	0.0846

6	0.05	40	20	55.43	0.9024	99.48	0.0584
7	0.05	40	20	61.15	0.9370	99.89	0.0635
8	0.05	40	20	94.52	0.8769	99.89	0.0404
9	0.05	40	20	115.50	0.9460	99.90	0.0527
6	0	40	20	0.75	0.9844	4.663	0.0321
6	0.01	40	20	1.99	0.9251	46.05	0.0431
6	0.03	40	20	20.66	0.9515	90.05	0.0522
6	0.05	40	20	55.43	0.9024	99.48	0.0584
6	0.07	40	20	55.37	0.9302	99.84	0.0594
6	0.05	10	20	4.98	0.9584	55.83	0.0236
6	0.05	20	20	10.53	0.9107	83.50	0.0258
6	0.05	30	20	41.18	0.9352	94.39	0.0451
6	0.05	40	20	55.43	0.9024	99.48	0.0584
6	0.05	50	20	68.32	0.9462	99.05	0.0590
6	0.05	40	10	87.99	0.9191	99.45	0.0453
6	0.05	40	20	55.43	0.9024	99.48	0.0584
6	0.05	40	30	33.57	0.9574	92.03	0.0576
6	0.05	40	40	15.25	0.9637	87.03	0.0673

S6. Reaction mechanism of chlorine evolution

The following three main reaction pathways in the process of electrode chlorine evolution are described.

Volmer-Tafel：

$$2Cl^- + 2* \rightarrow 2Cl^* + 2e^-$$

$$Cl^* + Cl^* \rightarrow 2* + Cl_2$$

Volmer-Heyrovsky：

$$2Cl^- + * \rightarrow Cl^* + e^- + Cl^-$$

$$Cl^* + Cl^- \rightarrow * + Cl_2 + e^-$$

Krishatalik：

$$2Cl^- + * \rightarrow Cl^* + e^- + Cl^-$$

$$Cl^* + Cl^- \rightarrow Cl^{*+} + e^- + Cl^-$$

$$Cl^{*+} + Cl^- \rightarrow * + Cl_2$$

where, * are the active sites (surface oxygen or metal atoms).

At the beginning, the reaction for electron loss of Cl^- happens, which then convert to Cl_2 on the surface of the PbO_2/PVDF/CC composite. Generally, the process of chlorine evolution comprises three main reaction pathways: Volmer-Tafel, Volmer-Heyrovsky, and Krishatalik, and the Volmer-Heyrovsky is regarded as the most likely pathway of single-crystalline transition metal oxides.

Reaction mechanism of chlorine electrooxidation ammonium is summarized as followed.

$$Cl_2 + H_2O \leftrightarrow HClO + H^+ + Cl^-$$

$$HClO \leftrightarrow H^+ + ClO^-$$

$$HClO + \frac{2}{3}NH_4^+ \rightarrow \frac{1}{3}N_2 + H_2O + \frac{5}{3}H^+ + Cl^-$$

$$4HClO + NH_4^+ \rightarrow NO_3^- + H_2O + 6H^+ + 4Cl^-$$

$$HClO + NH_4^+ \rightarrow NH_2Cl + H_2O + H^+$$

$$HClO + NH_2Cl \rightarrow NHCl_2 + H_2O$$

$$HClO + NHCl_2 \rightarrow NCl_3 + H_2O$$

$$H_2O + NHCl_2 \rightarrow NOH + 2H^+ + 2Cl^-$$

$$NOH + NHCl_2 \rightarrow N_2 + H^+ + Cl^-$$

$$NOH + NH_2Cl \rightarrow N_2 + H^+ + Cl^- + H_2O$$

For the indirect oxidation of active chlorine, the subsequent reaction is the formation of Cl_2 through the anode chlorine evolution reaction, and then Cl_2 dissolves in water and undergoes hydrolysis to generate HClO. Then, the free chlorine reacts with ammonium to produce gaseous nitrogen and a small amount of nitrate nitrogen. Also, ammonium will react with free chlorine to generate the mono-, di-, and tri-chloramine intermediates. Among them, NCl_3 has low solubility in water and cannot exist stably. In most cases, it is mainly in the forms of NH_2Cl and $NHCl_2$, and will further be converted to N_2.

References

55. Yang, H.Y; Chen, Z.L.; Guo, P.F.; Fei, B; Wu, R.B. B-doping-induced amorphization of LDH for large-current-density hydrogen evolution reaction. *Appl. Catal. B: Environ.* **2020**, *261*, 118240. https://doi.org/10.1016/j.apcatb.2019.118240
56. Zuo, S.J.; Zhang, Y.Q.; Guo, R.X.; Chen, J.Q. Efficient removal of ammonia nitrogen by an electrochemical process for spent caustic wastewater treatment. *Catalysts* **2022**, *12*, 121357. https://doi.org/10.3390/catal12111357.
